# Impact of the Pb and Zn ore mining industry on the pollution of the Biała Przemsza River, Poland

**DOI:** 10.1007/s10661-016-5233-3

**Published:** 2016-04-01

**Authors:** Magdalena Jabłońska-Czapla, Katarzyna Nocoń, Sebastian Szopa, Aleksandra Łyko

**Affiliations:** Institute of Environmental Engineering, Polish Academy of Sciences, 34 Skłodowskiej-Curie St, 41-819 Zabrze, Poland

**Keywords:** Water, Sediment, Biała Przemsza River, BCR, Heavy metals, Suspension, Mobility

## Abstract

The development of mining and metallurgic industries of Pb and Zn ores in the Biała Przemsza catchment area has had a strong influence on the condition of the surface water and bottom sediments. In the following study, total contents of metals and metalloids were researched in the water and bottom sediment samples from the Biała Przemsza River. The samples were collected monthly in 2014 at five sampling points along the river. The research helped to determine correlations between the parameters and components of the water environment (metals/metalloids, cations/anions, pH, Eh, conductivity, carbon (TOC, IC, TC), and suspension). The contents of metals and metalloids were determined with inductively coupled plasma-mass spectrometry (ICP-MS), whereas anions and cations were investigated with ion chromatography (IC). The simplified Community Bureau of Reference (BCR) three-step sequential chemical extraction was performed on Biała Przemsza River bottom sediments collected in April, July, and October. At its lower course, the Biała Przemsza River water did not meet the Polish surface water quality standards. The Biała Przemsza River water is mainly loaded with metals. Toxic concentrations of Cd, Pb, and Zn were observed at sampling points in Okradzionów and Sławków. The toxic Tl concentration was exceeded (2–6 μg/L) at three sampling points. The Biała Przemsza River bottom sediments were composed mostly of medium and fine sand. The BCR extraction of the bottom sediments demonstrated that Cd and Zn were bound to cations/anions and carbonates loosely adsorbed on the bottom sediments in spring and summer. Such a situation was observed at all the sampling points, except for BP3 in Okradzionów. The organic carbon concentration increased along the river course.

## Introduction

Heavy metal concentrations in river systems are often considered as indicators of anthropogenic influence. As heavy metals pose a risk to the natural environment, their concentrations need to be tracked and assessed. The assessment and effect of the sediment pollution can be reviewed by the trace metal partitioning in the sediment-water interface (Hejabi and Basavarajappa 2013). Researchers carried out a number of studies on the sediment pollution caused by anthropogenic or natural activities (Wardas et al. 1996; Ciszewski 2003; Johnson et al. 2005; Adekola and Eletta 2007; Aleksander-Kwaterczak and Helios-Rybiska 2009; Soares et al. 1999; Sakata et al. 2010; Wang et al. 2010; Giri and Singh 2014; Yang et al. 2015).

Heavy metal contents in water and bottom sediments are determined by the changing physicochemical parameters, such as pH (Loska and Wiechula 2000; Gundersen and Steinnes 2001), redox conditions (Jabłońska-Czapla et al. 2014), suspension content (Nocoń et al. 2013), or temperature (Kostecki 2004). Additionally, they are often influenced by other factors such as the changing year (Samarina 2003) or monsoon seasons (Sundaray et al. 2012).

Due to the long-term anthropogenic impact, industry, and other human activities on the Upper Silesia, its rivers are the source of contamination of other Polish regions. The Biała Przemsza River flowing through areas rich in zinc and lead ores, for centuries polluted by mining and metallurgical industry only to a small extent, retained its natural character (Lis and Pasieczna 1999; Pasieczna et al. 2010; Nocoń et al. 2012).

The exploitation of the Pb and Zn ores in the Biała Przemsza River catchment area began in the 16th century. Large-scale mining operations have been conducted there since the mid-20th century. The ore-bearing dolomites and overlying limestone form a productive aquifer. The zinc-lead deposits of the Upper Silesia-Cracow area, Poland, appear to be similar to Mississippi Valley-type (MVT) ores in carbonate rocks. The principal mines are 10 to 25 km northwest of Katowice in southwestern Poland. These ores, exploited for more than six centuries, account for a major part of total European production of zinc (Guilbert and Park 1986). Ore textures are varied but uniformly similar in the district (Sass-Gustkiewicz et al. 1982). Scientist noted that all of the ore is in original carbonate layers and that dolomitization and sulfide emplacement go hand in hand, and also that the ores are stratabound in distribution but not stratiform in textural detail. Because the ore is dominantly carbonate host rock, the local rock buffers potential acid mine and tailings water. Therefore, significant mobility of heavy metals is likely to be spatially restricted. In some districts, water from underground MVT mines serves as a local domestic water source (Upper Silesia, Poland). Most MVT deposits are in carbonate aquifers that can have enormous fluid transmissivity of regional extent. Many ore districts contain one or more sandstone aquifers that potentially allow greater contaminant dispersion than carbonate aquifers. Low iron oxide and clay contents of carbonate aquifers could permit greater dispersion of some elements at neutral or slightly alkaline pH (Leach et al. 2010).

The mine waters are separated underground with respect to their pollution degree. The most polluted mine waters, together with those from the settling pond, enter the Biała Przemsza River in its middle course (Ciszewski 2001). The drainage system of the river is made of natural tributaries under strong anthropogenic influence, as well as ditches and canals. The hydrogeological regime of the catchment area has been disturbed due to the drainage of the exploited mine deposits.

The metal transformations in the river system are dominated by at least five hydraulic mechanisms, i.e., mixing due to the particle size and density, chemical solution dispersion, Fe and Mn complexes, and biological capture, dilution with clean unpolluted sediments, and exchange with the floodplain deposits (Taylor and Kesterton 2002). The changes in the heavy metal concentrations caused by floods are diversified. High-magnitude floods may cause a considerable decrease in the mean concentration of heavy metals which can be transported along the river channel over the distance of (at least) a few dozen kilometers. Floods cause only a short-term increase in the heavy metal pollution in the highly polluted rivers with a stable pollution source.

Heavy metals are retained in sediments due to various mechanisms (ion exchange, adsorption, and precipitation or coprecipitation). Thus, the metals occur in different fractions. Many sequential extraction schemes using different chemical reagents and experiment conditions have been developed for determining heavy metal speciation (Jiang and Sun 2014). The sequential extraction procedures (based on the Tessier procedure or its different versions (Lasheen and Ammar 2009) have been applied to soils and sediments to fractionate metals with different extractants or reagents, which is done to obtain more useful information about metal bioavailability and mobility. In order to harmonize the methodology throughout the EU, and to improve the result comparability, the Community Bureau of Reference (BCR) devised a simple, three-stage sequential extraction protocol for the operationally defined fractionation of trace metals in soil and sediment samples (Tokalioglu et al. 2003).

Suspension (Szymczak and Galińska 2013) and contents of inorganic (Li et al. 2006) and organic (Gao et al. 2000, 2002; Correl et al. 2001; Zhu and Olsen 2014) carbon constitute other essential parameters used to describe the river condition. High concentrations of suspension often occur during floods. In the regions under strong anthroporessure observed an increasing level of the pollution with suspension (Nocoń and Kostecki 2005a, b). High heavy metal concentrations were observed in the Upper Silesia urban area surface waters, such as the Kłodnica River. The concentrations of Cd, Pb, and Zn in this river largely exceeded the values for the surface water quality classes valid during the time of the research. The Kłodnica River tributaries also demonstrated increased heavy metal concentrations in water. Adamiec (2003) and Helios-Rybicka et al. (2005) indicated heavy pollution of the Vistula and the Oder below the water inflows from the Upper Silesia urban area.

The following paper presents the results of research into the contents of metals/metalloids (V, Cr, Mn, Fe, Co, Ni, Cu, Zn, Ga, As, Rb, Sr, Ag, Cd, Sb, Te, Ba, Tl, Pb, and U), ions (F^−^, Cl^−^, SO_4_^2−^, Na^+^, K^+^, Ca^2+^), suspension, and carbon in the Biała Przemsza River water and bottom sediments. The research objective was to determine the mobility and seasonal changes of the metal and metalloid contents in the river ecosystems of the Upper Silesia urban area using the example of the Biała Przemsza River, which is highly polluted by the Pb and Zn ore mining and processing industries.

## Methods

### Researched area

**Fig. 1:**
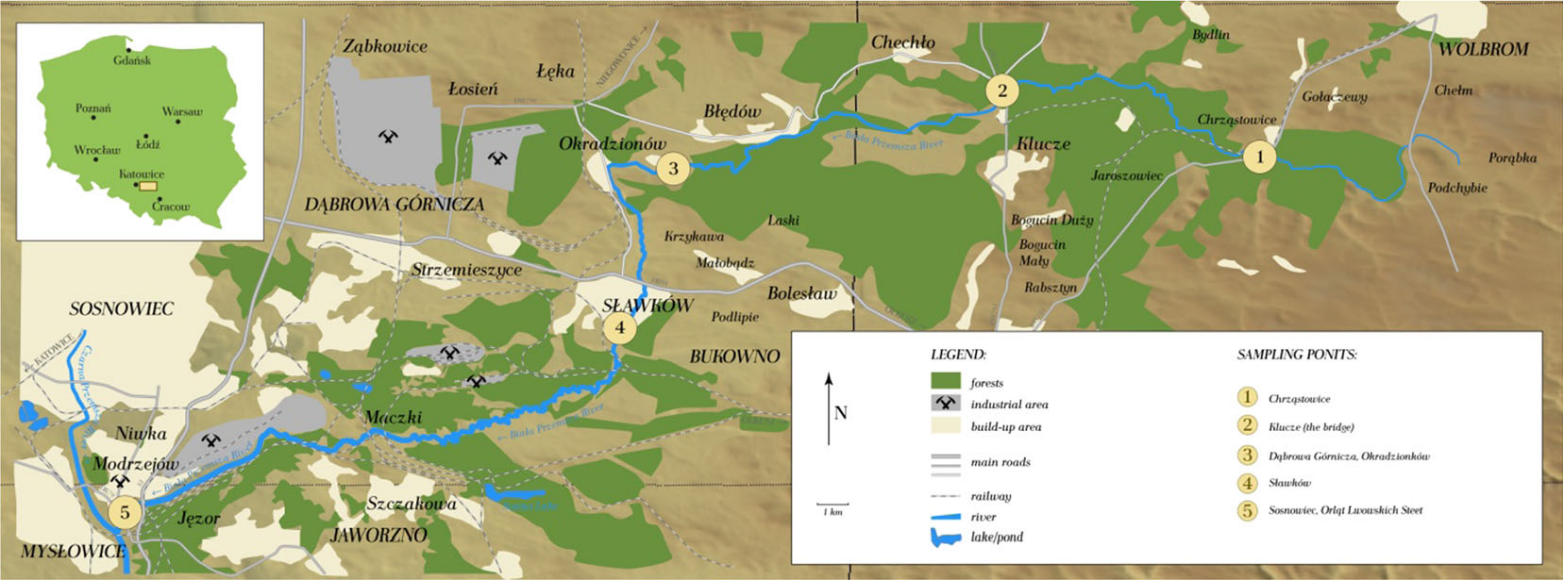
Location of the sampling points along the Biała Przemsza River

The Biała Przemsza River flows through the Lesser Poland and Silesian voivodships (Fig. 1). These areas are rich in zinc and lead ores. Ores from the Biała Przemsza catchment area are similar to those of the Mississippi Valley, especially those from Tennessee. The Silesian-Cracow lead and zinc deposits occur mostly within the so-called ore-bearing dolomites of the Middle Triassic (Musehelkalk) and are classified as Mississippi Valley-type deposits by the same authors (Sass-Gustkiewicz et al. 1982; Wodzicki 1987). Mississippi Valley-type (MVT) lead-zinc deposits are a varied family of epigenetic ore deposits that form predominantly in dolostone and in which lead and zinc are the major commodities. MVT deposits are most intensely studied in North America (Leach et al. 2010).

The river begins its course in the central part of the Polish Jura, i.e., in the Olkuska Upland. Then, flows through Błędowska Desert and in the vicinity of the Dąbrowa Górnicza flows through swampy areas, which are the most inaccessible, wild, and inhabited by unique species of fauna and flora. The Biała Przemsza River changes its character after the Biała River, the largest tributary flows into it. The majority of Biała River water is mine water from the Pomorzany Pb and Zn ore mine. The rest comes from the wastewater treatment plant in Olkusz. This tributary heavily pollutes the Biała Przemsza River, particularly in terms of heavy metals and suspension. In Okradzionów, the Biała Przemsza River radically changes its direction to the south. It starts meandering in Sławków and it gradually changes its direction to the southwest. After, Sławków river flows west far away from the urbanized areas. The next Biała Przemsza tributary is the Sztoła River, which comes from the Pb and Zn ore mine. In the “Three Emperors’ Corner” in Mysłowice, the Biała Przemsza River flows into the Przemsza River (Pasieczna et al. 2010; Nocoń et al. 2012).

### Sampling

Water and bottom sediment samples were collected monthly (April–December 2014) at five sampling points. The points were located in Chrząstowice (BP1), Klucze Osada (BP2), Dąbrowa Górnicza Okradzionów (BP3), and Sławków (BP4). The final point (BP5) was located at Orląt Lwowskich Street in Sosnowiec (very close to the “Three Emperors’ Corner”). The sampling points are shown in Fig. 1.

### Sample preparation

Directly after sampling in situ, basic physicochemical parameters (pH, redox potential (Eh), conductivity, and temperature) were analyzed. Directly after transport into the laboratory, the water samples to be analyzed with inductively coupled plasma-mass spectrometry (ICP-MS) were acidified with spectra pure nitric acid. Afterward, they were filtered through a 0.22-μm PES syringe filter. Next, the samples were analyzed to determine total analyte contents with an ICP-MS spectrometer. Bottom sediment samples were air-dried, sieved through a sieve (2-mm mesh), ground in a mortar, and mineralized in the MarsX microwave mineralizer. The bottom sediment mineralization was optimized with the certified reference material (NCS DC 733309). The mixture of 5 ml of HNO_3_ 65 %, 3 ml of HF 40 %, and 2 ml of H_2_O_2_ was applied. Metals and metalloids were determined in the obtained mineralizates with the ICP-MS spectrometer. Sediments collected in April (spring), July (summer), and October (autumn) 2014 were subjected to sieve analysis.

### Research methodology

The investigations were carried out with ICP-MS, ion chromatography (IC), and simplified BCR three-step sequential chemical extraction of the bottom sediments. The in situ physicochemical data (temperature, pH, electrolytic conductivity (EC), Eh) were collected using the CX-401 multiparameter meter (Elmetron, Poland) equipped with an ERH 111 glass electrode (Hydromet, Poland), an ERPt-111 platinum electrode (Hydromet, Poland), and a CD-2 conductometric sensor (Hydromet, Poland) with a built-in thermometer. EC and pH were double-checked and the pH meter was calibrated with three pH buffer solutions (pH = 3.0, 7.0, and 9.0).

The contents of selected metals and metalloids were determined with ICP-MS. The ICP-MS Elan 6100 DRC-e (Perkin Elmer, USA) spectrometer was used. The apparatus equipment and optimalization was carried out as in previous study (Jabłońska-Czapla 2015).

An application enabling the measurements of ^51^V, ^53^Cr, ^55^Mn, ^57^Fe, ^59^Co, ^60^Ni, ^65^Cu, ^66^Zn, ^69^Ga, ^75^As, ^85^Rb, ^88^Sr, ^107^Ag, ^114^Cd, ^121^Sb, ^130^Te, ^138^Ba, ^205^Tl, ^208^Pb, and ^238^U isotopes was prepared. Standards, blanks, and samples were measured using ^103^Rh as internal standard (10 μg/L, Merck, Germany). Solution of 10 μg/L Rh was introduced into all solutions on line, by second tubing on peristaltic pomp. All solutions of multielemental (Merck, Germany) and 1 g/L single-element standards of Cd, Pb, Zn, and Sb (Merck, Germany) were prepared daily by the dissolution reference materials (Merck, Germany) in water obtained from Milli-Q System (Millipore, USA) and used for the calibration. The calibration was verified with another certified multielement standard, i.e., XXI (Merck, Germany). The method validation was performed on the basis of the certified reference material (NIST 1643e Trace Elements in Water). The ionic composition of the samples (F^−^, Cl^−^, SO_4_^2−^, Na, K, Ca) was determined with the Metrohm IC system (Metrohm, Switzerland) equipped with IC 818 pump, IC 837 eluent degasser, IC 830 interface, IC 820 separation module, and IC 819 conductivity detector.

Organic, inorganic, and total carbon was determined with the carbon analyzer. Dissolved organic carbon (DOC) was measured with the TOC-5000A carbon analyzer (Shimadzu, Japan). Total organic carbon (TOC) was determined with the infrared spectroscopy. The calculations were made with the differential method. Suspension was determined in accordance with the standard (PN-EN 872: 2007). The sieve analysis was performed according to the Polish standard (PKN-CEN ISO/TS 17892-4: 2009). Additionally, the way and form in which Mn and other metals were bound in the bottom sediments were determined with the simplified BCR three-step sequential chemical extraction (Tokalioglu et al. 2003). The extraction procedure conditions are given in previous work (Jabłońska-Czapla 2015).

## Results and discussion

### Physicochemical parameters

**Fig. 2:**
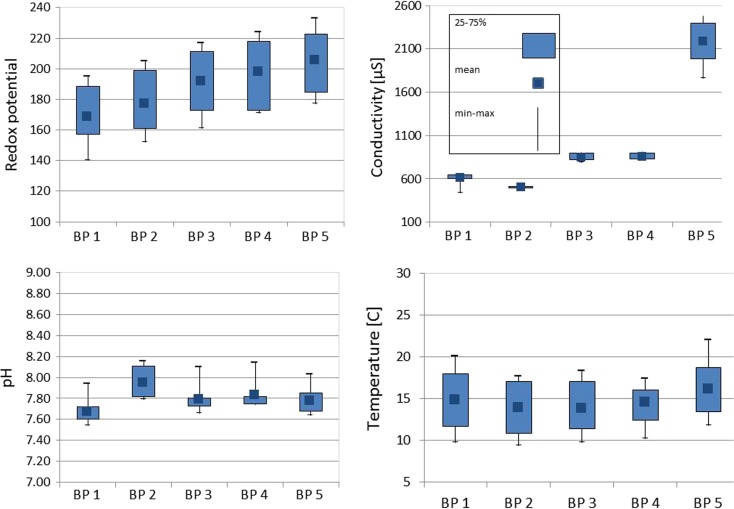
Box plots of pH, conductivity (μS), temperature (°C), and redox potential (Eh) data for Biała Przemsza river monitoring (five sampling points BP1–BP5, samples taken from April to December 2014 Biała Przemsza River water)

The basic physicochemical parameters are presented in Fig. 2. The pH values were the lowest in the first sampling point in Chrząstowice, right at the source of the river, and then rapidly grew, so that the highest value was obtained in Klucze. In subsequent sampling points from BP3 to BP5 (in the course of the river), water pH decreased. Drop in pH led to the dissolution of carbonates and hydroxides, and the metal ions were displaced with the hydrogen ions. The change in the redox potential caused the change in the metal-binding forms in the solid phase and the pH drop, which increased metal mobility (Forstner 1986). Similarly, the redox potential increased with the river course, to obtain the highest value in the last sampling point in Sosnowiec. The highest conductivity value was observed at BP5, where the highest ion concentrations were found (principally chlorides and sulfates). Temperature is an important parameter when it comes to the release of metals and metalloids from the river bottom sediments (Tsai et al. 2003). In the case of the Biała Przemsza River, this parameter increased with the river course and, like conductivity, gets the highest value in BP5.

pH values did not drop below 7 in any case. It is rather unlikely that such small pH variations will have an effect on the water, since these values cannot support the necessary acidity to produce carbonate dissolution nor trace element mobility (Zn, Cu, Cd, Pb). On the contrary, especially As and Cr are elements with higher mobility at high pH values (Nriagu and Nieboer 1988; Bissen and Frimme 2003; Jabłońska-Czapla et al. 2014).

### Metal, metalloid, and ion contents in the Biała Przemsza River water

The minimum and maximum average concentrations of metal and metalloid as well as limits in surface water regulated by law are shown in Table 1.

**Table 1:** Basic statistics on metals and metalloids in the studied waters, detection limits (LOD), expanded uncertainty (U), and guideline values established by the WHO (WHO 2008) and Polish (Dz.U 2014) and earlier (Dz.U 2004a, b) (for third water quality class) legislative bodies (PL) for surface water

Analytes (μg/L)	Number of samples	LOD	U (%)	Mean	Median	Min	Max	SD	PL (2014)	PL (2004)	WHO
V	45	0.09	10.00	1.17	0.85	0.30	4.29	0.89	50.00	–	–
Mn	45	0.033	21.08	140.15	156.34	13.09	326.66	83.81	–	500.00	40.00
Co	45	0.002	10.00	1.20	1.37	0.13	3.92	0.89	50.00	–	–
Ni	45	0.024	10.00	7.68	9.05	1.97	14.48	4.25		50.00	70.00
Cu	45	0.064	12.37	1.77	1.64	0.40	3.22	0.76	50.00	60.00	2000.00
Zn	45	0.181	34.65	438.63	307.00	11.14	1636.29	481.65	100.00	100.00	–
As	45	0.096	27.81	3.36	3.44	0.28	6.74	2.02	50.00	50.00	–
Rb	45	0.003	10.00	54.12	3.83	1.84	383.37	107.59	–	–	–
Sr	45	0.008	10.62	318.76	209.55	68.50	1296.60	313.05	–	–	–
Ag	45	0.002	14.05	0.03	0.01	0.00	0.24	0.05	5.00	–	–
Cd	45	0.04	11.38	1.62	0.95	0.06	6.76	1.93	–	1.00	3.00
Te	45	0.002	12.65	1.20	1.00	0.59	3.07	0.58	–	–	–
Ba	45	0.01	16.96	128.35	88.20	58.24	484.05	99.20	500.00	500.00	–
Tl	45	0.002	15.18	2.10	2.72	0.01	5.15	1.80	2.00	–	–
Pb	45	0.036	27.98	56.63	29.27	0.41	509.83	85.17	–	20.00	10.00
Fe	45	0.09	31.9	794.43	758.56	223.51	1328.63	296.70	–	1000.00	–
Ga	45	0.01	15.50	3.26	2.85	1.58	6.48	1.26	–	–	–
U	45	0.01	11.39	0.54	0.49	<0.01	1.07	0.32	–	–	15.00
Cr	45	0.013	11.44	2.96	1.85	0.26	13.41	3.13	50.00	50.00	50.00
Sb	45	0.05	10.92	0.49	0.43	0.18	1.51	0.25	2.00	–	20.00

*LOD* limit of detection, *U* expanded uncertainty, *SD* standard deviation, *PL* (2014) values established by Polish legislation (Dz.U. 2014, *PL (2004)* values established by Polish legislation (Dz.U 2004a, b, *WHO* values established by (WHO 2008)

**Fig. 3:**
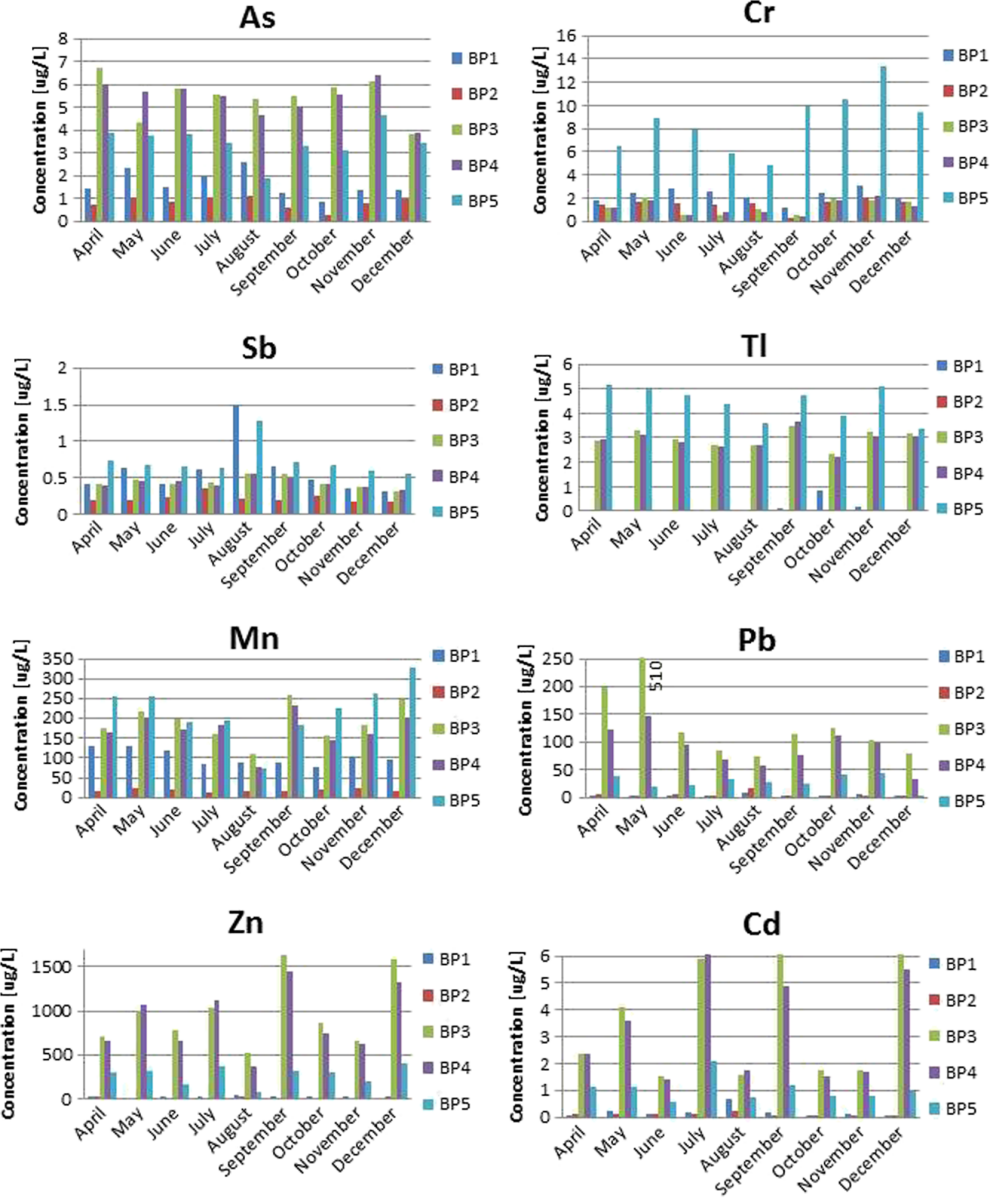
As, Sb, Cr Tl, Mn, Zn, Cd, and Pb concentrations in the Biała Przemsza River water samples. Sampling points: *BP1*—Chrząstowice; *BP2*—Klucze Osada; *BP3*—Dąbrowa Górnicza Okradzionów; *BP4*—Sławków; *BP5*—Sosnowiec

As it is shown in Fig. 3, the highest Cr concentration was observed at BP5 (nearly 14 μg/L). The highest As concentrations were found in Sławków and Dąbrowa Górnicza–Okradzionów. The Bobrza River located in the western part of the Świętokrzyskie Mountains is characterized by lower levels of chromium average 3.80 μg/L (Rabajczyk 2011).

The highest Sb concentrations in Biała Przemsza River water were measured at BP1 and BP5. As these analyte contents were low, it would be possible to assign the Biała Przemsza River water to Class I (Dz.U 2011) of the surface water quality.

Nevertheless, low trace concentrations of As, Sb, and Cr were accompanied by high contents of Mn, Pb, Cd, and Zn. The Biała Przemsza River catchment area is known from significantly developed metallurgic and mining industry. The largest industrial facility in the Biała Przemsza River catchment area is the ZGH “Bolesław” (Olkusz area), known for the Pb and Zn ore exploitation. For this reason, in the Sławków and Dąbrowa Górnicza areas, Biała Przemsza River is heavily polluted by Pb, Cd, and Zn.

In September, high Mn contents were accompanied by high Cd and Zn contents. The lowest Mn concentration was found in Klucze. A slightly higher one was observed at BP1 in Chrząstowice (about 100 μg/L). It increased until December 2014, when it reached 326 μg/L at BP5 (Sosnowiec) (Jabłońska-Czapla 2015). Due to the total Mn content in the Biała Przemsza River water, it can be classified within classes II and below II of the surface water quality. In terms of the Mn content, the water quality was very good (class I water quality) (Dz.U 2004a) only at BP2 (Klucze). Polich-Latawiec et al. (2014) showed that the mean Mn content in the Sztoła River water (the left-bank Biała Przemsza River tributary) was 30–40 μg/L. The Sztoła River like Biała River pollutes the Biała Przemsza River especially with metals and metalloids. Both tributaries supplied the mine water from the Pomorzany Pb and Zn ore mine, wastewater from the ZGH “Bolesław” in Bukowno and wastewater treatment plant in Olkusz. For that reason, the Biała Przemsza River water became seriously polluted with heavy metals and other elements below their mouths. The mean Mn, Pb, and Zn contents observed in the lower course water of the Biała Przemsza River were 160, 35, and 540 μg/L, respectively (Pasieczna et al. 2010).

The content of heavy metals in Utrata River is lower (Mn = 20 μg/L, Pb = 35 μg/L, and Zn = 40 μg/L) (Wojtkowska 2011). Metal concentration in the Odra River water varied in wide ranges: up to 10.9 μg/L of Pb, 1.26–202 of Zn, and 0.82–334 of Mn (Helios-Rybicka et al. 2005).

In Poland, only some rivers are characterized by class I purity of water and often in only parts of the river. Some of the cleanest rivers in Poland are mountain rivers such as Dunajec River. In Dunajec River, the majority of waters met the requirements for class I water purity and only Zn and Cr concentrations exceeded permissible values for this class. Contents of all elements fell within the Al quality category waters, except in incidental cases of exceedance of Cr recommended value (Wiśniowska-Kielian and Niemiec 2004).

The Zn contents in the Biała Przemsza River varied largely. At Chrząstowice, the river water was assigned to class I, but in Dąbrowa Górnicza and Sławków, it was given class III–IV (Dz.U 2004a). A similar situation was observed for Cd, the Biała Przemsza River water could be classified within classes I–III (Dz.U 2004a) of the surface water quality. Due to large Pb contents, which significantly exceeded 50 μg/L, the river water could not be assigned to any class. The highest Pb, Zn, and Cd contents were observed at BP3 and BP4. Such a situation was likely caused by the Biała Przemsza River tributaries such as the Biała River.

**Fig. 4:**
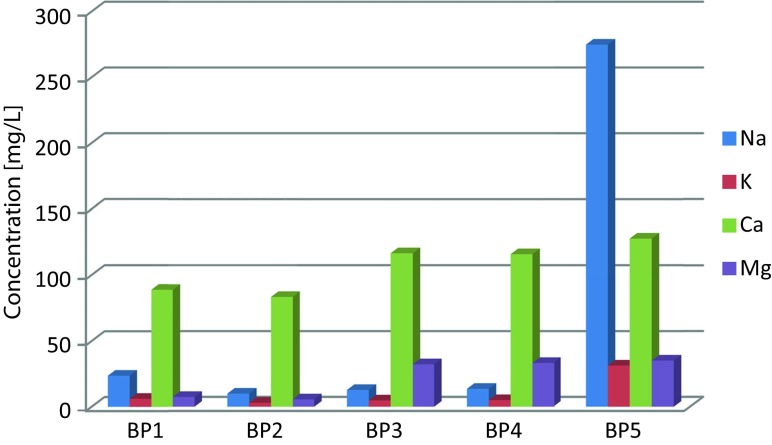
Mean concentrations (April–December 2014) of cations in the Biała Przemsza River water samples

High concentrations of metals and metalloids were accompanied by the increased anion contents. The highest concentration of chlorides (and consequently of Na) was observed at BP5. The sulfate contents significantly increased between BP3 and BP5. The comparison of the cation contents (Fig. 4) clearly indicates the growing trend in the metal contents along the river course. The maximum sulfate and chloride contents were 361.4 and 359.2 mg/L, respectively (Table 2). Another “controversial” element is toxic Tl. As it is shown in Fig. 6, the Tl concentrations at BP3–BP5 were high (2–6 μg/L). In relation to the Polish legal regulations for Tl (2 μg/L), the observed values largely exceeded the permissible levels. Tl accompanies the Pb and Zn ores. It is harmful to organisms and demonstrates strong genotoxic activity (Kabata-Pendias and Pendias 1999). At BP1 and BP2, the Tl concentrations did not exceed 1 μg/L.

**Table 2:** Basic statistics on the major components in the studied waters, and guideline values established by the WHO (WHO 2008), EU (2003), and Polish (Dz.U 2004b, 2007) legislative bodies (PL) for drinking water

Ions	LOD	Number of samples	Mean	Median	Min	Max	S.D.	PL	EU	WHO
F^−^ (mg/L)	0.06	24	0.4	0.2	<0.06	2.1	0.6	5	5	1.5
Cl^−^ (mg/L)	0.01	24	86.3	20.1	10.5	392.9	133.4	–	–	250
NO_3_^−^ (mg/L)	0.03	24	13.2	12.7	6.9	22.6	3.3	50	50	50
SO_4_^2−^ (mg/L)	0.33	24	158.6	185.7	30.7	361.4	102.1	–	–	500
Na^+^ (mg/L)	0.09	24	67.8	13.7	8.9	359.2	109.0	–	–	200
K^+^ (mg/L)	0.09	24	10.2	5.6	2.5	44.9	11.2	–	–	–
Mg^2+^ (mg/L)	0.17	24	107.8	110.3	54.5	164.6	23.8	–	–	–
Ca^2+^ (mg/L)	0.22	24	23.7	27.0	3.2	53.3	15.2	–	–	–

*LOD* limit of detection

### Carbon and suspended sediment in the Biała Przemsza River water

**Fig. 5:**
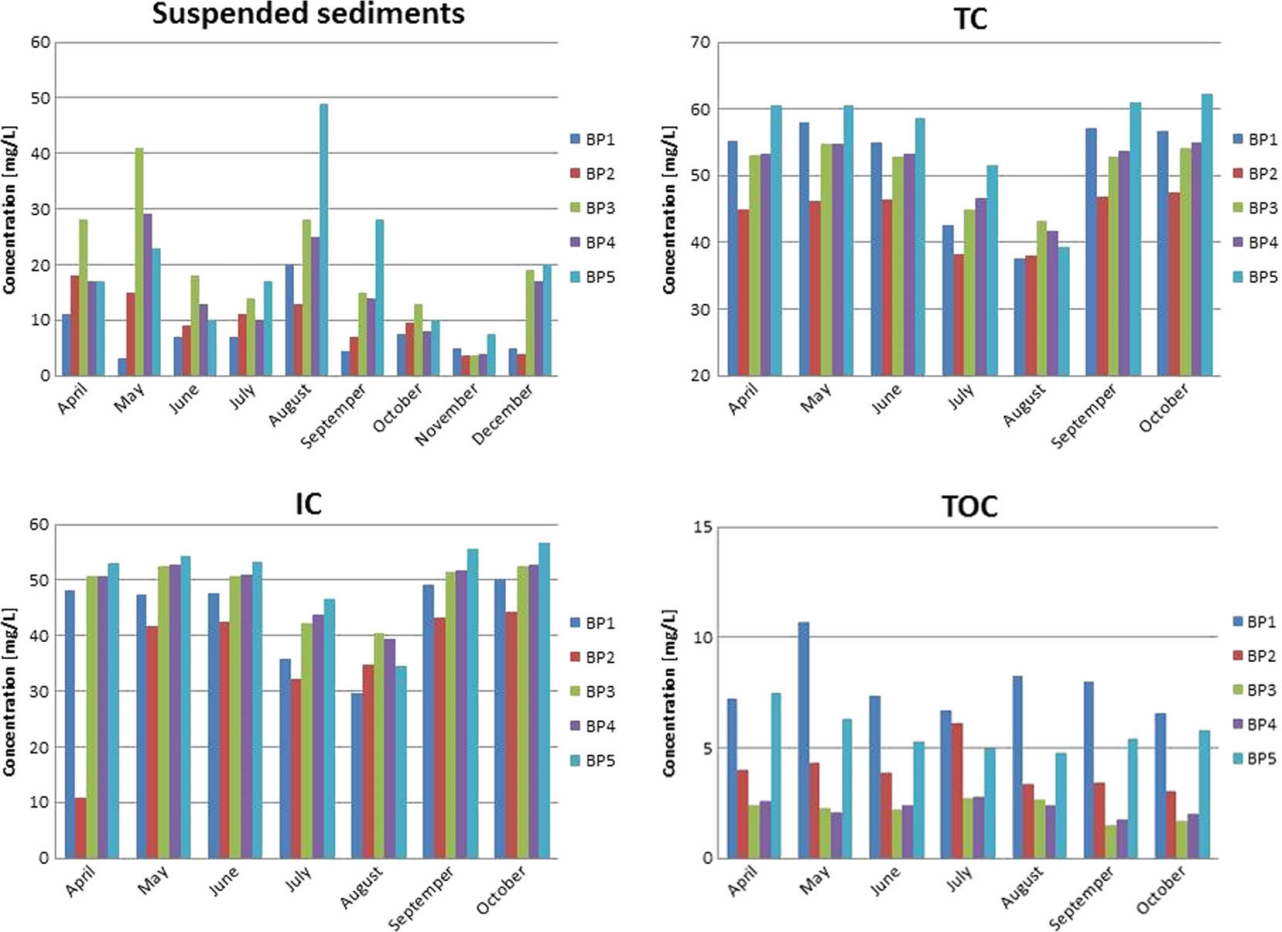
Suspension, total carbon (TC), total organic carbon (TOC), and inorganic carbon (IC) concentrations in the Biała Przemsza River water samples. Sampling points: *BP1*—Chrząstowice; *BP2*—Klucze Osada; *BP3*—Dąbrowa Górnicza Okradzionów; *BP4*—Sławków; *BP5*—Sosnowiec

The suspended sediment content in the Biała Przemsza River varied highly. However, it demonstrated a general trend consisting in the suspension concentration increase in May and August 2014. The suspension content largely depended on the sampling point location (Fig. 5). The highest concentration was observed at BP5 (Sosnowiec) in August 2014, whereas the lowest one was measured at BP1 (Chrząstowice, close to the riverhead) in May 2014. The researchers observed a gradual increase in the suspension concentration caused by the anthropogenic activity (highly developed Pb and Zn ore mining and processing industries) influencing the condition of the surface water and bottom sediments. The suspension content was correlated with the Mn content, particularly at BP5 (coefficient of determination *R*^2^ = 0.5905).

**Fig. 6:**
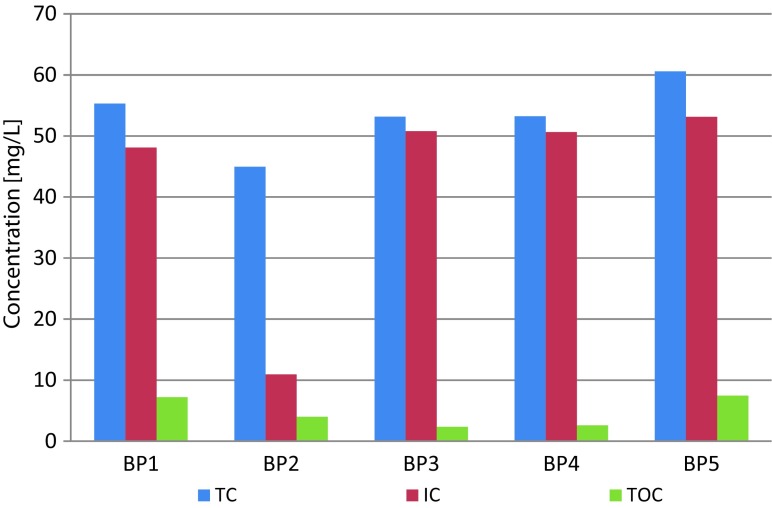
Organic, inorganic, and total carbon contents in the Biała Przemsza River water samples

Similarly to suspension, there were strong correlations between total, inorganic, and organic carbon contents and the Mn content at BP5 (the *R*^2^ values of 0.7687, 0.7124, and 0.5960, respectively). At the remaining sampling points, there was a medium-strong correlation between the contents of Mn and total and inorganic carbon. Due to the TOC content, which is a factor characterizing the oxygen conditions and organic pollutants, the Biała Przemsza River water quality can be classified within class I (Dz.U 2014). The maximum TOC concentration was 10.68 mg/L. It was observed at BP1. There was a slightly increasing trend in the organic carbon concentration along the river course. The only exception was BP2 (Klucze), at which the TOC, inorganic carbon (IC), and total carbon (TC) concentrations were the lowest. Figure 6 presents the comparison of the carbon form contents in the Biała Przemsza River. Inorganic carbon was the main component of total carbon.

### Total metal and metalloid contents in the bottom sediments

Table 3 presents the sieve analysis results. It shows that the Biała Przemsza River bottom sediments chiefly contain medium and fine sand (particle fraction of 0.5–0.2 mm).

**Table 3:** Sieve analysis of the Biała Przemsza River bottom sediments according to the standard (PKN-CEN ISO/TS 17892-4:^2009^)

Month	Sampling point	>2 mm	2–1 mm	1–0.5 mm	0.5–0.2 mm	0.2–0.1 mm	<0.1 mm	Unit
April	BP1	0.16	0.22	15.79	77.85	3.64	0.17	%
	BP2	0.29	0.17	15.54	73.77	10.06	0.18	%
	BP3	5.51	11.94	12.47	31.89	25.85	12.34	%
	BP4	0.00	0.01	2.27	88.87	8.78	0.08	%
	BP5	0.07	0.23	2.00	80.85	15.44	1.41	%
July	BP1	0.16	0.22	15.79	77.85	3.64	0.17	%
	BP2	2.43	2.45	6.32	63.08	23.17	3.68	%
	BP3	1.30	0.75	1.02	36.36	34.93	26.94	%
	BP4	0.00	0.00	0.39	78.43	20.22	0.96	%
	BP5	1.26	1.48	8.77	80.18	7.55	0.76	%
October	BP1	0.07	0.11	9.25	84.21	6.09	0.28	%
	BP2	11.97	3.19	5,74	38.08	31.42	9.60	%
	BP3	0.08	0.00	0.92	59.91	33.03	6.06	%
	BP4	0.18	0.11	0.31	32.10	30.28	37.02	%
	BP5	0.52	0.76	4.46	82.38	10.82	1.05	%

*N* number of samples, *LOD* limit of detection, *SD* standard deviation

**Fig. 7:**
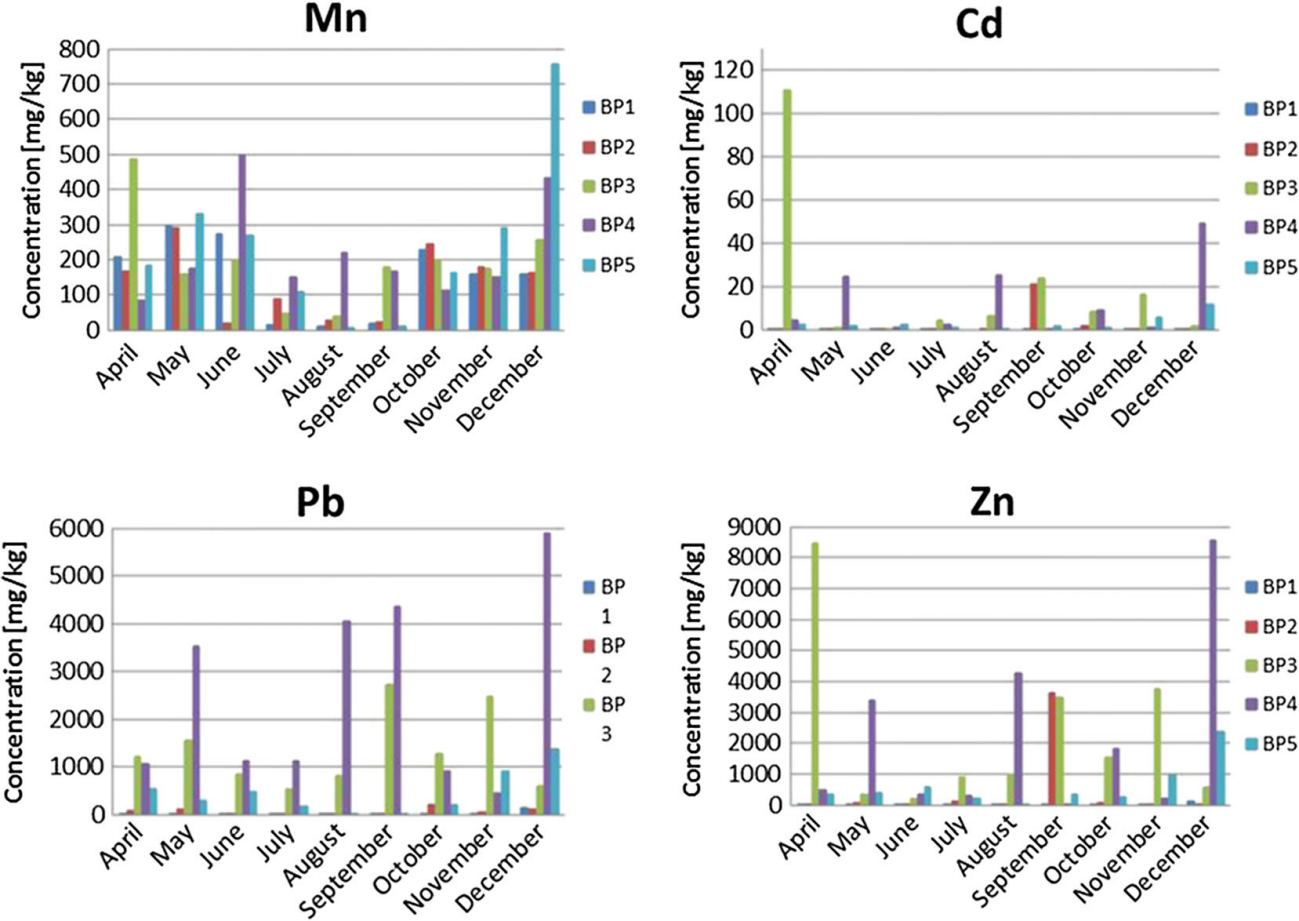
Total Mn, Cd, Pb, and Zn concentrations in the Biała Przemsza River bottom sediments. Sampling points: *BP1*—Chrząstowice; *BP2*—Klucze Osada; *BP3*—Dąbrowa Górnicza Okradzionów; *BP4*—Sławków; *BP5*—Sosnowiec

Figure 7 presents total Mn, Pb, Zn, and Cd contents in the Biała Przemsza River. Nocoń et al. (2012) investigated the Biała Przemsza River bottom sediments and obtained the highest Mn contents in Sławków and Okradzionów. The following study confirmed these findings. The highest Mn contents were observed at the sampling points in Sławków and Sosnowiec. The maximum Mn concentration was determined at BP5 in December 2014 (nearly 760 mg/kg). When comparing the total Mn content in the Biała Przemsza River bottom sediments, it must be said that high Mn contents (mean value of 2727 mg/kg) were also found in the Baba Stream bottom sediments (supplying the Sztoła River) (Lis and Pasieczna 1999). As many authors indicated, the Biała Przemsza River bottom sediments contained large Cd, Pb, Zn, and Tl contents. The Tl concentration value was even 9.41 mg/kg (BP3 at Okradzionów in April 2014). The Pb concentrations were particularly high at BP3 and BP4 (even 6000 mg/kg in December 2014). The highest Cd, Pb, and Zn concentrations were observed at BP3 and BP4. Such a situation was caused by the Biała Przemsza River tributaries. The Biała River, which flows in the Biała Przemsza just ahead of BP3 in Okradzionów, does not have natural sources at present, or they appear seasonally. The majority of the Biała River water is constituted by the mine water from the Pomorzany Pb and Zn ore mine, discharged through the Dąbrówka canal. The remaining part comes from the wastewater treatment plant in Olkusz and small tributaries (a number of water fluxes on the left river side). The Biała River heavily pollutes the Biała Przemsza River, particularly in terms of heavy metals and suspension.

The highest As and Sb concentrations in the bottom sediments were measured at BP3 and BP4 in April 2014. In the same month, a very high Cr content was observed at BP4 (Sławków).

Lecce and Pavlowsky (2001) studied the Blue River located on the northern margin of the Upper Mississippi Valley Pb-Zn District in the Driftless Area of southwestern Wisconsin, USA. This region was one of the most important sources of Pb and Zn ore in the USA. The Blue River sediments have high Zn concentrations due to the close proximity of this site to the mine source. All of sampling locations record a period with low Zn concentrations (300–600 mg/kg) followed by a distinct Zn peak (8000–15,000 mg/kg). After the peak mining period, concentrations fall to about 2000–3000 mg/kg. The results indicate clearly the similarities of the Mississippi Valley Pb and Zn with the Biała Przemsza River basin. In terms of Zn content, the Biała Przemsza sediments are characterized by high concentrations of Zn from 58–9427 mg/kg. Mała Wełna River (western Poland) sediments comprise up to 90 mg/kg (Frankowski et al. 2008). Bottom sediments of the Utrata River were characterized by high content of heavy metals. Content range (in mg/kg) was as follows: for zinc 71–338, copper 8.2–281, lead 49.6–111.6, and cadmium 1.25–4.39, respectively (Wojtkowska 2011).

In the Kłodnica River sediments, concentrations of lead, copper, and zinc were indicative of moderate contamination, whereas the concentration of cadmium showed that the bottom sediments of the Klodnica were severely contaminated. The rise observed in the iron and manganese content of the bottom sediments was associated with the wastewater discharge from coal mines (Barbusiński and Nocoń 2011). The Kłodnica River is tributary of the Odra River. Sediments of the Odra River are polluted by metals in wide ranges (mg/kg): 6.39–192 As, 1.22-21.7 Cd, 29–427 Pb, 23.5–89.3 Ni, 20.7–400 Cr, 34.1–298 Cu, 77.6–3113 Zn, 329–6020 Mn (Helios-Rybicka et al. 2005).

Ciszewski et al. (2008) detected postdepositional changes in the concentration of zinc, cadmium, lead, and manganese in vertical profiles of alluvial sediments in the Upper Odra River, southern Poland. Depending on the depth concentrations, Zn, Pb, Cd, and Mn were on average 950, 450, and 6 mg/kg, respectively.

Other authors (Bojakowska and Sokołowska 1996) studied the Bystrzyca River flows across the Lublin Upland and falls into the Wieprz River. They noted very high concentration of cadmium occurring along the river stretch. Some samples exhibit Cd concentration as high as 500 mg/kg.

### BCR three-step sequential chemical extraction of the bottom sediments

**Fig. 8:**
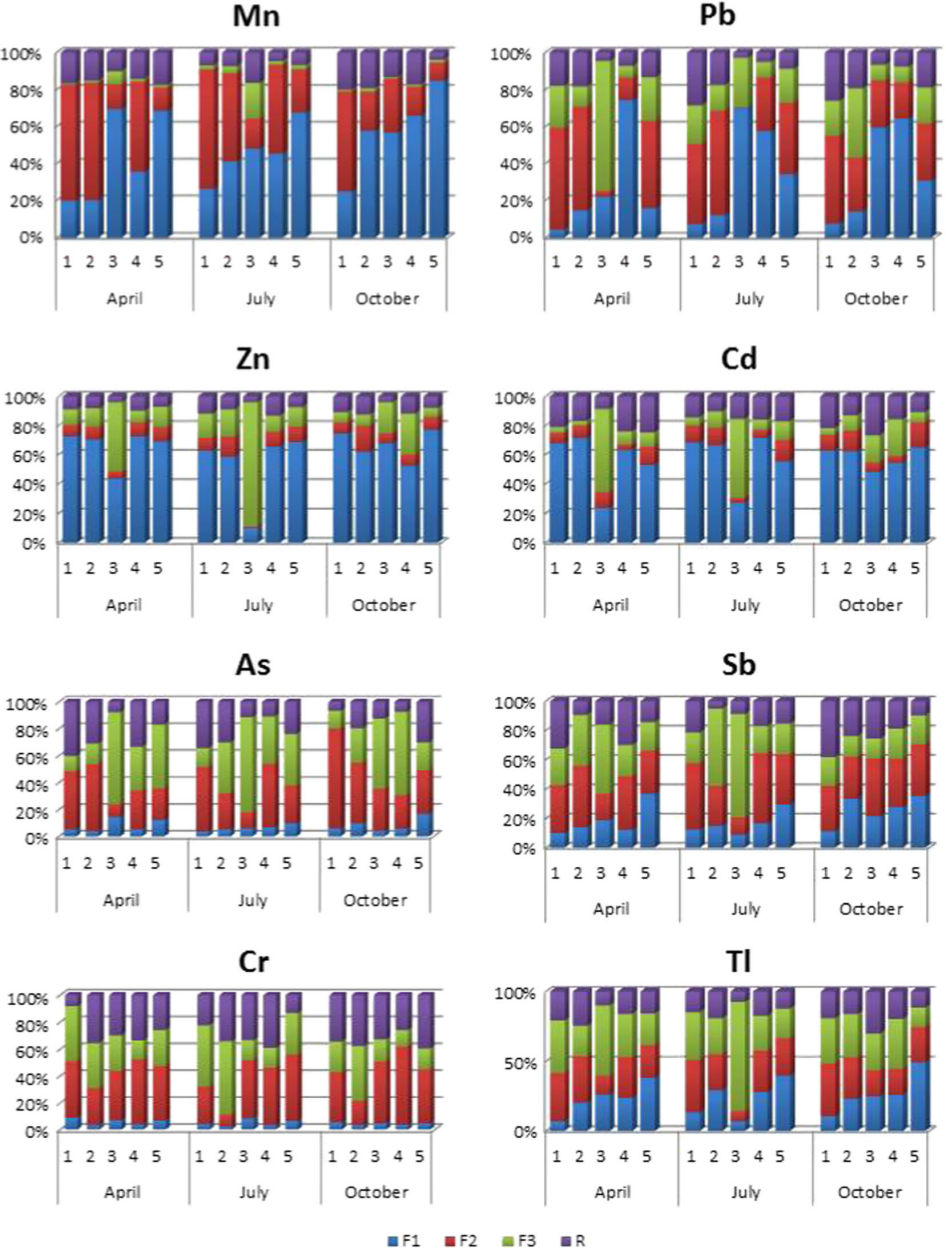
Simplified BCR three-step sequential extraction for Mn, Pb, Zn As, Sb, Cr, Tl, and Cd. F1—ion-exchanged and carbonated fraction; *F2*—oxide fraction; *F3*—organic matter and sulfide fraction; R-residual fraction. Sampling points: *BP1*—Chrząstowice; *BP2*—Klucze Osada; *BP3*—Dąbrowa Górnicza Okradzionów; *BP4*—Sławków; *BP5*—Sosnowiec

The BCR analysis (Fig. 8) demonstrated that Mn was mainly bound to Mn and Fe oxides at BP1 and BP2, where the Biała Przemsza River demonstrated its mountainous character. Starting from BP3 (Dąbrowa Górnicza-Okradzionów), Mn was bound to ions and carbonates. Additionally, the largest content of Mn bound to organic matter was found at BP3. The sequential chemical extraction of the bottom sediments showed that Cd and Zn were mostly bound to cations/anions and carbonates loosely adsorbed on the bottom sediments in spring and summer. Such a situation was observed at all the sampling points, except for BP3 in Okradzionów. This sediment had the largest contents of cadmium and zinc bound to sulfides and organic matter.

The sequential chemical extraction also demonstrated that arsenic and chromium were bound to the oxide and sulfide and organic matter fractions in the Biała Przemsza River bottom sediments. At BP1, arsenic was mainly bound to oxides in all seasons (Jabłońska-Czapla 2015). For antimony, there were seasonal changes in the ion exchange fraction percentage. It increased during the year and the highest value was found at BP5. Sequential leaching techniques are useful for estimating potential remobilization of toxic metals from polluted solid material. One of these procedures, which has become the most generally accepted in the study of aquatic particulate matter, was originally designed by Tessier et al. (1979). The data from a large number of leaching experiments on dredged materials (Chester et al. 1984) suggest that the surplus of metal contaminants, introduced into the aquatic system by human activities, usually exists in relatively unstable chemical form. They should, therefore, be more accessible for short- and middle-term geochemical processes (including biological uptake) than the detrital, predominantly. Species differentiation can be used in estimates of remobilization of metals under changing environmental conditions. Forstner (1986) studying the sediments came to the conclusion that in the estuarine environment, the “exchangeable fraction” will be particularly affected. However, changes of pH and redox potential could also influence other weakly held phases (e.g., carbonate and manganese oxides). Lowering of pH will affect the “exchangeable,” then the “easily reducible;” and then parts of the “moderately reducible” fractions, with the latter consisting of Fe oxyhydrates in less crystallized forms.

Referring to the work data contained in Table 4, (Forstner 1986) obtained results which indicate that existing pH and redox conditions promote the release of Zn, Cd, and Pb from sediments into the waters of the Biała Przemsza River. However, in the case of chromium associated mainly with oxides, sequential chemical extraction has confirmed that a neutral environment and slightly oxidizing conditions favored demobilization of Cr.

**Table 4:** Relative mobilities of elements in sediments and soils as a function of Eh and pH (Forstner 1986)

Relative mobility	Electron activity		Proton activity	
	Reducing	Oxidizing	Neutral alkaline	Acid
Very low mobility	Al, Cr, Mo, V, U, Se, S, B, Hg, Cu, Cd, Pb	Al, Cr, Fe, Mn	Al, Hg, Cr, Cu, Ni, Co	Si
Low mobility	Si, K, P, Ni, Zn, Co, Fe	Si, K, P, Pb	Si, K, P, Pb, Fe, Zn, Cd	K, Fe(III)
Medium mobility	Mn	Co, Ni, Cu, Hg, Zn, Cd	Mn	Al., Pb, Cu, Cr, V
High mobility	Ca, Na, Mg, Sr	Ca, Na, Mg, Sr, Mo, V, U, Se	Ca, Na, Mg, Sr	Ca, Na, Mg, Sr, Zn, Cd, Hg, Ni, Co, Mn
Very high mobility	Cl, I, Br	Cl, I, Br, S, B	Cl, I, Br, S, B, Mo, V, U, Se	Cl, I, Br, S, B

## Summary and conclusions

The research showed that the Biała Przemsza River water did not meet the standards at its lower course. It also demonstrated significant differences in the contents of specific components in the river. The Biała Przemsza River water is mostly loaded with metals. Its pH ranged between 7.54 and 8.16, whereas the conductivity values were 437–2,480 μS. The mean Pb and Cd contents were 56.6 and 1.62 μg/L, respectively. Such findings classified the river water (taking into account the classification of the old Polish regulation from 2004) within class V (for Pb) and class IV (for Cd) of the surface water quality (Dz.U 2004a, b). The research also revealed high Tl contents. Its mean value was 2.1 μg/L, which classified the Biała Przemsza River water (Dz.U 2014) below class II of the surface water quality.

In the Biała Przemsza River water samples, there were no significant seasonal variations in concentrations of the analytes. Only in the case of antimony were the highest concentrations obtained in August. In the case of lead, the highest concentrations were noted in spring. The concentration of heavy metals in the Biała Przemsza River waters is characterized by a strong dependence on sampling point. The highest concentrations of analytes were found in the last three collection points: in Dabrowa Górnicza, Slawków, and Sosnowiec. The concentration of lead in the sediment was the highest in the summer months, when it recorded the highest temperature of air and water. Furthermore, in December, it reported extremely high concentrations of lead of nearly 6000 mg/kg. Lead was associated with ion-exchanged and carbonated fraction especially in two sampling points, BP3 and BP4. The most mobile elements of the Biała Przemsza River sediments are lead and zinc. These elements associated mainly with the ion-exchanged and carbonated fraction only in July at Dąbrowa Górnicza sampling point were associated with organic matter. Chromium concentration in the occurring conditions of pH and redox was characterized by low mobility of sediments and showed no seasonal changes. Only in the case of thallium was there a gradual increase in the concentration of this element in sediments along the river. The highest share of thallium in organic matter and sulfide fraction was recorded in July in Dąbrowa Górnicza. Dissolved heavy metal forms are transported into the bottom sediments during sorption and other biochemical processes. Consequently, the water quality improves, but metal concentrations in the sediments increase. The heavy metal content in the sediments is a good indicator for the pollution level of the water environment. The Biała Przemsza River is the last river in the Upper Silesian urban area which (in part course) still maintains its natural character despite centuries of mining exploitation in its catchment area. The mining works have caused the irreversible changes in the water environment. Nevertheless, the natural area in the river vicinity has been preserved and constitutes one of the most valuable areas in Upper Silesia.
